# Design and construction of a pyrolysis unit – demonstrated in connection with gas chromatography-ion mobility spectrometry

**DOI:** 10.1016/j.ohx.2026.e00769

**Published:** 2026-04-02

**Authors:** Martin Lippmann, Moritz Hitzemann, Ayke Eckhardt, Daniel Röckrath, Stefan Zimmermann

**Affiliations:** Leibniz University Hannover, Institute of Electrical Engineering and Measurement Technology, Department of Sensors and Measurement Technology, Appelstr. 9a, 30167 Hannover, Germany

**Keywords:** Pyrolizer, Curie temperature, Curie point, Gas chromatography, Ion mobility spectrometry, GC, IMS, GC-IMS

## Abstract

In analytical and bioanalytical chemistry, pyrolysis is often used for thermal decomposition of complex, non-volatile samples into smaller and more volatile constituents in an inert atmosphere as a preparatory step in various analytical techniques to reveal the composition of a sample by quantifying its constituents and building blocks, rendering it a powerful tool for analyzing samples ranging from synthetic polymers to natural biomaterials. This work presents an open-source pyrolysis unit with temperature regulation based on the Curie temperature of a ferromagnetic wire serving as the sample holder. The system’s modular design makes it adaptable to many different analytical applications. Depending on the voltage of the power supply and the excitation frequency of the coil, fast temperature rise times of 2 s can be reached, and the temperature is selectable between 350 °C and 1100 °C, depending on the material used for the ferromagnetic wire. Coupling this pyrolyzer to a gas chromatograph-ion mobility spectrometer for analyzing probiotics demonstrates one exemplary application.

## Graphical abstract

**Figure:**
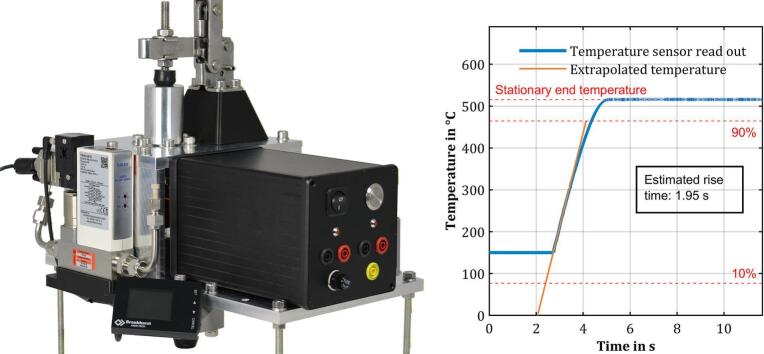

## 1 Specifications table

**Table:** 

Hardware name	Curie point pyrolysis unit
Subject area	• Chemistry and biochemistry • General
Hardware type	• Measuring physical properties and in-lab sensors • Biological sample handling and preparation
Closest commercial analog	Curie Point Pyrolyzer JHP-10 by Japan Analytical Industry Co., Ltd.
Open source license	CC BY 4.0
Cost of hardware	Approx. 4.670 €
Source file repository	https://doi.org/10.17632/btgp48659m.1

## 2 Hardware in context

In analytical and bioanalytical chemistry, analyzing complex, non-volatile organic samples is standard. Pyrolysis, a process that thermally decomposes such organic samples into smaller and more volatile constituents in an inert atmosphere, is often used as a preparatory step to reveal the composition of a sample by quantifying its constituents and building blocks by different analytical techniques such as gas chromatography-mass spectrometry (GC–MS) or gas chromatography-ion mobility spectrometry (GC-IMS) [1], [2], [3], [4], [5], [6], [7], [8], [9].

Despite its importance, the high cost and complexity of most commercially available pyrolysis equipment can be a significant barrier, particularly for educational institutions, small laboratories, and research facilities with limited budgets. This presents a clear need for the development of an affordable and accessible alternative that provides the necessary functionality for thorough analytical investigations. To date, no open-source version of an analytical pyrolysis unit has been widely recognized or adopted. However, such an open-source pyrolysis unit could address the need by providing a scalable and modular solution, leveraging readily available materials and detailed construction guidelines. Such a unit enables a wide range of applications, from the characterization of polymers and environmental samples to the study of biomass for bioenergy research or the investigation of bacteria and potentially viruses [10], [11], [12], [13]. Furthermore, it supports forensic analysis and the breakdown of pollutants in environmental samples [4].

This article presents the design, construction, and validation of an open-source pyrolysis unit. It aims to equip laboratories with the tools necessary to perform high-quality analytical research without the burden of high costs.

## 3 Hardware description

A pyrolysis unit rapidly heats a solid or liquid sample to a desired pyrolysis temperature in an inert atmosphere, at which the sample breaks down to smaller, more volatile molecules. This process is used in analytical chemistry to analyze the composition of, *e.g.*, organic materials [4], [14]. The basic structure of a pyrolysis unit is generally similar, regardless of the heating method employed. The most important component of a pyrolysis unit is the heating chamber, also known as the reaction chamber, which must be gas-tight to prevent oxygen or other reactive species from reaching the sample and should have a minimal dead volume. Large dead volumes result in a prolonged time required to flush the system at a constant gas flow, causing broader peaks in gas chromatography (GC) when directly feeding the GC column with the pyrolyzate. This, in turn, reduces GC separation efficiency. Moreover, dead volumes can serve as storage sites for sample residues, increasing the risk of cross-contamination and necessitating cleaning processes that raise the operating costs of the pyrolysis unit.

The pyrolysis process begins with the application of the sample onto a sample holder, which can be, for example, a heat-resistant wire, a quartz crucible, a glass tube, or a metal cup. The sample is then introduced into the reaction chamber, where the sample holder and sample are heated to the preset pyrolysis temperature. The exact ramp-up time and the adjustable final temperature depend on the type and design of the pyrolysis unit. A neutral carrier gas, such as nitrogen or argon, transports the constituents of the pyrolyzed sample in the gas phase for further analysis. The pyrolysis products are typically analyzed using gas chromatography combined with mass spectrometry or, in some cases, also ion mobility spectrometry. Since a GC typically has a low flow rates of only a few milliliters per minute, sample sizes ranging from a few micrograms to a tenth of a microgram are sufficient.

A rapid temperature ramp-up is advantageous for two reasons: firstly, it minimizes possible secondary reactions, and secondly, it allows the generation of short gas plugs consisting of the constituents of the pyrolyzed sample that can be directly injected into the GC separation column. Shorter gas plugs typically yield better GC separation of the pyrolyzate. Therefore, a fast and reproducible temperature ramp or heating time is crucial. Additionally, the temperature should be maintained at a constant level for the desired duration of the pyrolysis process. A reproducible heating process ensures consistent measurement conditions, resulting in the reproducible generation of pyrolysis constituents from a sample. Various methods exist to heat samples to the point where chemical bonds break. Three primary heating methods have emerged: inductive heating (utilizing the Curie point), resistive heating (using glow wires), and pyrolysis ovens [4], [7]. Inductive heating usually using the Curie point method employs a high-frequency induction coil to generate a magnetic field, which heats a ferromagnetic sample holder through hysteresis and eddy current losses [15]. Above the Curie temperature, ferromagnetic properties and hysteresis losses diminish, self-regulating to the material's specific Curie temperature [16], [17]. It benefits from self-regulating temperature without external measurement, but is limited to specific Curie temperatures. Cobalt (Co) has the highest Curie temperature among all pure elements, at 1130 °C [18]. The Curie temperature of iron (Fe) is 770 °C [19], [20], and that of nickel (Ni) is 358 °C [21], [22]. In contrast, resistive heating employs a conductive wire through which an electric current flows, generating heat to raise the sample to the desired temperature. The heating process is precisely controlled by an external regulator, ensuring accurate temperature management. This method allows flexible temperature end points across a wide operational range and can also achieve rapid heating rates. It is beneficial for analyzing samples across a broad temperature range, but requires precise regulation and direct connection of the wire to a power source [4]. Opposite to the previous options, using a pyrolysis oven, the entire reaction chamber is heated to an isothermal state, and samples are introduced once the desired temperature is reached. This method simplifies sample introduction and does not require specific heating ramp control, but it can lead to variable sample temperatures and potential secondary reactions due to the large internal volume of the chamber. Each method has its strengths and limitations regarding temperature control, sample preparation, and analytical flexibility, with trade-offs between complexity, cost, and operational range. However, results of different pyrolysis methods are comparable [23], [24], [25].

The implementation of the pyrolysis unit presented in this work focuses on reproducibility, heating ramp-up time, and cost. Therefore, a Curie point-based pyrolysis unit was chosen because it requires substantially less complexity in temperature regulation. Its temperature is controlled based on a physical effect. Considering additional uncertainties, such as the longer and indeterminate heating time of samples in pyrolysis ovens, it can be concluded that the Curie point method achieves higher reproducibility with less effort, but it comes with less flexibility in setting pyrolysis temperature. For the requirements described in this work, this disadvantage is outweighed by its advantages compared to the other concepts. This led to a pyrolysis unit offering the following benefits:

• *User-Friendly: Operates with just one button, reducing the need for extensive training and allowing for quick setup.*
• *Compact Design: Saves space in the lab, making it easy to fit into existing setups without major rearrangements.*
• *Compatibility: Easily integrates with various GC and other analytical devices without requiring additional software or modifications.*
• *Costs: Offers a budget-friendly alternative to commercially available devices without sacrificing performance.*
• *Versatile Use: Supports both standard and novel laboratory tasks.*

## 4 Design files summary

### 4.1 Schematics

The schematic files.SchDoc,.PcbDoc,.PrjPCB and.PrjPcbStructure as listed in Table 1 contain all information regarding the electronics to drive the pyrolysis unit. To directly order a PCB, the Gerber files and NC Drill files are included, which simplifies the ordering process and eliminates the need for specialized software. The “Curie_Point_Pyrolyzer_Schematics.pdf” contains all schematics and the PCB layout as well.

**Table 1:** List of design files required to build the pyrolysis unit.

Design file name	File type	Open source license	Location of the file
Curie_Point_Pyrolyzer.PrjPcb	Schematics	CC BY 4.0	Mendeley Data
Curie_Point_Pyrolyzer.PrjPcbStructure	Schematics	CC BY 4.0	Mendeley Data
Overview.SchDoc	Schematics	CC BY 4.0	Mendeley Data
PowerSupply.SchDoc	Schematics	CC BY 4.0	Mendeley Data
FrequencyGeneration.SchDoc	Schematics	CC BY 4.0	Mendeley Data
FullBridge.SchDoc	Schematics	CC BY 4.0	Mendeley Data
Peripherie.SchDoc	Schematics	CC BY 4.0	Mendeley Data
CoilControl.PcbDoc	Schematics	CC BY 4.0	Mendeley Data
Curie_Point_Pyrolyzer_Schematics.pdf	Schematics	CC BY 4.0	Mendeley Data
CoilControl.apr	GerberFiles	CC BY 4.0	Mendeley Data
CoilControl.EXTREP	GerberFiles	CC BY 4.0	Mendeley Data
CoilControl.GBL	GerberFiles	CC BY 4.0	Mendeley Data
CoilControl.GBO	GerberFiles	CC BY 4.0	Mendeley Data
CoilControl.GBP	GerberFiles	CC BY 4.0	Mendeley Data
CoilControl.GBS	GerberFiles	CC BY 4.0	Mendeley Data
CoilControl.GM1	GerberFiles	CC BY 4.0	Mendeley Data
CoilControl.GM2	GerberFiles	CC BY 4.0	Mendeley Data
CoilControl.GP1	GerberFiles	CC BY 4.0	Mendeley Data
CoilControl.GP2	GerberFiles	CC BY 4.0	Mendeley Data
CoilControl.GTL	GerberFiles	CC BY 4.0	Mendeley Data
CoilControl.GTO	GerberFiles	CC BY 4.0	Mendeley Data
CoilControl.GTP	GerberFiles	CC BY 4.0	Mendeley Data
CoilControl.GTS	GerberFiles	CC BY 4.0	Mendeley Data
CoilControl.REP	GerberFiles	CC BY 4.0	Mendeley Data
CoilControl-macro.ARP_LIB	GerberFiles	CC BY 4.0	Mendeley Data
CoilControl.DRR	NC Drill Files	CC BY 4.0	Mendeley Data
CoilControl.LDP	NC Drill Files	CC BY 4.0	Mendeley Data
CoilControl-Plated.TXT	NC Drill Files	CC BY 4.0	Mendeley Data
Parameter.xlsx	CAD file	CC BY 4.0	Mendeley Data
Bottom_adapter.ipt	CAD file	CC BY 4.0	Mendeley Data
Bottom_flange.ipt	CAD file	CC BY 4.0	Mendeley Data
Bottom_plate.ipt	CAD file	CC BY 4.0	Mendeley Data
Centering_rod.ipt	CAD file	CC BY 4.0	Mendeley Data
Copper_coil.ipt	CAD file	CC BY 4.0	Mendeley Data
CPP.iam	CAD file	CC BY 4.0	Mendeley Data
CuriePointPyrolyzer.ipj	CAD file	CC BY 4.0	Mendeley Data
Draht.ipt	CAD file	CC BY 4.0	Mendeley Data
Fuß_Gehäuse.ipt	CAD file	CC BY 4.0	Mendeley Data
Flow_controller_mount.ipt	CAD file	CC BY 4.0	Mendeley Data
Knob_inner_part.ipt	CAD file	CC BY 4.0	Mendeley Data
Knob_outer_part.ipt	CAD file	CC BY 4.0	Mendeley Data
Liner.ipt	CAD file	CC BY 4.0	Mendeley Data
O-Ring_Liner.ipt	CAD file	CC BY 4.0	Mendeley Data
Side_plate.ipt	CAD file	CC BY 4.0	Mendeley Data
Spannzange.ipt	CAD file	CC BY 4.0	Mendeley Data
Spring.ipt	CAD file	CC BY 4.0	Mendeley Data
Toggle_clamp_mounting_block.ipt	CAD file	CC BY 4.0	Mendeley Data
Threaded_bar.ipt	CAD file	CC BY 4.0	Mendeley Data
Top_adapter.ipt	CAD file	CC BY 4.0	Mendeley Data
Top_plate.ipt	CAD file	CC BY 4.0	Mendeley Data
Bottom_adapter.stp	CAD file	CC BY 4.0	Mendeley Data
Bottom_flange.stp	CAD file	CC BY 4.0	Mendeley Data
Bottom_plate.stp	CAD file	CC BY 4.0	Mendeley Data
BRIGHT_Unit_mount.stp	CAD file	CC BY 4.0	Mendeley Data
Flow_controller_mount.stp	CAD file	CC BY 4.0	Mendeley Data
Knob_inner_part.stp	CAD file	CC BY 4.0	Mendeley Data
Knob_outer_part.stp	CAD file	CC BY 4.0	Mendeley Data
PCB_mount_1.stp	CAD file	CC BY 4.0	Mendeley Data
PCB_mount_2.stp	CAD file	CC BY 4.0	Mendeley Data
Side_plate.stp	CAD file	CC BY 4.0	Mendeley Data
Top_adapter.stp	CAD file	CC BY 4.0	Mendeley Data
Top_plate.stp	CAD file	CC BY 4.0	Mendeley Data
Bottom_adapter.pdf	Technical Drawing	CC BY 4.0	Mendeley Data
Bottom_flange.pdf	Technical Drawing	CC BY 4.0	Mendeley Data
Bottom_plate.pdf	Technical Drawing	CC BY 4.0	Mendeley Data
Enclosure.pdf	Technical Drawing	CC BY 4.0	Mendeley Data
Enclosure_plate1.pdf	Technical Drawing	CC BY 4.0	Mendeley Data
Enclosure_plate2.pdf	Technical Drawing	CC BY 4.0	Mendeley Data
Knob_inner_part.pdf	Technical Drawing	CC BY 4.0	Mendeley Data
Knob_outer_part.pdf	Technical Drawing	CC BY 4.0	Mendeley Data
Side_plate.pdf	Technical Drawing	CC BY 4.0	Mendeley Data
Top_adapter.pdf	Technical Drawing	CC BY 4.0	Mendeley Data
Top_plate.pdf	Technical Drawing	CC BY 4.0	Mendeley Data

### 4.2 CAD files

All required components to build or order the mechanical part of the pyrolysis unit are provided as.ipt and.iam files. Ready-to-use files for 3D printing are available as.stl files. To simplify the ordering of mechanical parts from external manufacturers, the technical drawings are also included as.pdf files.

## 5 Bill of materials summary

The bill of materials as presented in Table 2 can also be found in the external file “bill_of_materials.xlsx” on Medeley Data (https://doi.org/10.17632/btgp48659m.1). Prices in brackets are bulk prices. Table 3 shows optional components required for operation with an external flow controller and adjustable excitation frequency.

**Table 2:** Bill of materials for assembly of the pyrolysis unit.

Designator	Component	Number	Cost per unit −currency	Total cost-currency	Source of materials	Material type
Ferromagnetic wire	Ferromagnetic wire	1	235.00 €	235.00 €	Sigma Aldrich	Metal
Bottom_plate	Bottom plate	1	123.00 €	123.00 €	Mechanical supplier	Metal
Top_plate	Top plate	1	79.00 €	79.00 €	Mechanical supplier	Metal
Side_plate	Side plate	2	38.00 €	76.00 €	Mechanical supplier	Metal
Knob_outer_part	Knob outer part	1	96.64 €	96.64 €	Mechanical supplier	Metal
Knob_inner_part	Knob inner part	1	109.24 €	109.24 €	Mechanical supplier	Metal
Top_adapter	Top adapter	1	138.66 €	138.66 €	Mechanical supplier	Metal
Bottom_adapter	Bottom adapter	1	117.65 €	117.65 €	Mechanical supplier	Metal
Bottom_flange	Bottom flange	1	126.05 €	126.05 €	Mechanical supplier	Metal
Liner	Liner	1	42.86 €	42.86 €	BGB Analytik	Glass
PCB_mount_1	PCB mount 1	1	n.a.	n.a.	Your own 3D print	Polymer
PCB_mount_2	PCB mount 2	1	n.a.	n.a.	Your own 3D print	Polymer
5 V Fan	5 V Fan	2	14.83 €	29.66 €	RS Components	Non-specific
Fan grill	Fan grill	2	2.22 €	4.44 €	RS Components	Metal
Toggle clamp	Toggle clamp	1	51.26 €	51.26 €	RS Components	Metal
TC_mounting_block	TC mounting block	2	n.a.	n.a.	Your own 3D print	Polymer
Threaded bar	Threaded bar	4	1.79 € (15.02 €)	7.16 €	RS Components	Metal
Collet chuck	Collet chuck	1	7.13 €	7.13 €	Conrad	Metal
Spring	Spring	1	0.52 € (5.19 €)	0.52 €	RS Components	Metal
Rubber cap for M8 screw	Rubber cap for M8 screw	1	1.96 € (9.81 €)	1.96 €	RS Components	Polymer
6x2 O-ring	6x2 O-ring	1	0.22 € (5.47 €)	0.22 €	RS Components	Polymer
12x2 O-ring	12x2 O-ring	1	0.28 € (6.99 €)	0.28 €	RS Components	Polymer
17x2 O-ring	17x2 O-ring	1	0.37 € (9.22 €)	0.37 €	RS Components	Polymer
20x2 O-ring	20x2 O-ring	1	0.40 € (9.98 €)	0.40 €	RS Components	Polymer
30x2 O-ring	30x2 O-ring	1	0.73 € (7.25 €)	0.73 €	RS Components	Polymer
M3x8	M3x8	6	0.33 € (19.77 €)	1.99 €	RS Components	Metal
M3x12	M3x12	4	0.32 € (18.76 €)	1.26 €	RS Components	Metal
M3x25	M3x25	4	0.43 € (25.30 €)	1.70 €	RS Components	Metal
M3 washer	M3 washer	18	0.03 € (7.91 €)	0.48 €	RS Components	Metal
M3 hexagon nut	M3 hexagon nut	8	0.04 € (9.68 €)	0.31 €	RS Components	Metal
M4x12	M4x12	1	0.36 € (18.19 €)	0.36 €	RS Components	Metal
M4x20	M4x20	2	0.47 € (23.66 €)	0.95 €	RS Components	Metal
M6x20	M6x20	4	0.44€ (21.86 €)	1.75 €	RS Components	Metal
M6x25	M6x25	9	0.51 € (25.52 €)	4.59 €	RS Components	Metal
M6x35	M6x35	15	0.68 € (33.87 €)	10.16 €	RS Components	Metal
M6x40	M6x40	2	0.81 € (40.67 €)	1.63 €	RS Components	Metal
M6x70	M6x70	4	1.19 € (29.77 €)	4.76 €	RS Components	Metal
M6 washer	M6 washer	68	0.04 € (11.07 €)	2.99 €	RS Components	Metal
M6 hexagon nut	M6 hexagon nut	32	0.09 € (21.72 €)	2.78 €	RS Components	Metal
LED socket	LED socket	2	1.25 €	2.50 €	Conrad	Non-specific
Cable bushing	Cable bushing	2	0.63 €	1.26 €	Conrad	Polymer
Pressure switch	Pressure switch	1	9.24 €	9.24 €	Conrad	Non-specific
6,3 A Fuse	6,3 A Fuse	1	0.57 €	0.57 €	Reichelt	Metal
Fuse holder	Fuse holder	1	1.67 €	1.67 €	Conrad	Non-specific
Rocker switch	Rocker switch	1	2.34 €	2.34 €	Conrad	Non-specific
Safety banana socket red	Safety banana socket red	2	1.67 €	3.34 €	Conrad	Non-specific
Safety banana socket black	Safety banana socket black	2	1.68 €	3.36 €	Conrad	Non-specific
Safety banana socket green/yellow	Safety banana socket green/yellow	1	1.67 €	1.67 €	Conrad	Non-specific
Universal aluminium enclosure	Universal aluminium enclosure	1	41.17 €	41.17 €	Conrad	Metal
3 mm LED red	3 mm LED red	2	0.10 €	0.20 €	Conrad	Semiconductor
Rubber screw on bumper	Rubber screw on bumper	4	1.50 €	6.00 €	Conrad	Polymer
Wire	Wire	1	22.30 €	22.30 €	Conrad	Non-specific
Copper wire	Copper wire	1	26.53 €	26.53 €	RS Components	Metal
C200, C201, C400, C401, C402, C403, C404, C405	3216 SMD-capacitor 10uF 50 V X5R	8	0.34 €	2.75 €	Mouser	Non-specific
C202, C203, C204, C411, C412, C419, C420	2012 SMD-capacitor 10uF 35 V X5R	7	0.27 €	1.87 €	Mouser	Non-specific
C300, C301, C302, C303, C500	2012 SMD-capacitor 10uF 35 V X7R	5	0.63 €	3.16 €	Mouser	Non-specific
C410, C417, C418, C425	3225 SMD-capacitor 470pF 100 V C0G	4	0.33 €	1.31 €	Mouser	Non-specific
C413, C415, C421, C423	1608 SMD-Feedthrough capacitor 0.47uF 6,3V X7S	4	0.10 €	0.40 €	Digikey	Non-specific
C414, C416, C422, C424	2012 SMD-capacitor 1nF NP0	4	0.45 €	1.79 €	Mouser	Non-specific
C406, C407, C408, C409	Electrolytic capacitor 470uF 100 V	4	2.89 €	11.55 €	RS Components	Non-specific
R200, R304	2012 SMD-resistor 430R	2	0.10 €	0.21 €	Mouser	Non-specific
R402, R409	2012 SMD-resistor 0R	2	0.09 €	0.18 €	Mouser	Non-specific
R300	2012 SMD-resistor 47 K	1	0.24 €	0.24 €	Mouser	Non-specific
R301	Carbon potentiometer	1	0.24 €	0.24 €	Reichelt	Non-specific
R400, R404, R407, R411	2512 Shunt 10R	4	0.56 €	2.24 €	Mouser	Non-specific
R401, R403, R408, R410	2012 SMD-resistor 1R	4	0.20 €	0.80 €	Mouser	Non-specific
R405, R406, R412, R413	2012 SMD-resistor 470 K	4	0.15 €	0.61 €	Mouser	Non-specific
D200	Silicon carbide power Schottky diode 650 V, 12 A	1	0.21 €	0.21 €	Mouser	Semiconducter
D300, D301	Standard PN-diode	2	0.08 €	0.15 €	Digikey	Semiconducter
D400, D401	Schottky barrier diode	2	0.13 €	0.27 €	Mouser	Semiconducter
IC300	IC GATE AND 2CH	1	0.20 €	0.20 €	Digikey	Semiconducter
IC301	Dual Schmitt Trigger Inverter	1	0.62 €	0.62 €	Mouser	Semiconducter
U300	Dual Schmitt Trigger Buffer	1	0.11 €	0.11 €	Mouser	Semiconducter
MP400, MP401	M4 Wire-to-Board connection	2	2.41 €	4.82 €	Mouser	Metal
P200	4 mm banana socket red	1	2.71 €	2.71 €	Mouser	Non-specific
P201	4 mm banana socket black	1	2.81 €	2.81 €	Mouser	Non-specific
P300, P500, P501	Pin Header	1	0.21 €	0.21 €	Reichelt	Metal
P502	SMA End Launch	1	9.30 €	9.30 €	Digikey	Metal
Q400, Q401, Q402, Q403	GaNFET	4	4.29 €	17.18 €	Digikey	Semiconducter
Q500	MOSFET	1	0.28 €	0.28 €	Digikey	Semiconducter
S200	Fuse holder	1	1.92 €	1.92 €	Mouser	Non-specific
U400, U401	Half-Bridge GaN Driver	2	3.11 €	6.22 €	Digikey	Semiconducter
Y300	Voltage Controlled Silicon Oscillator	1	4.91 €	4.91 €	Digikey	Semiconducter
Heat-conducting paste	Heat-conducting paste	1	18.54 €	18.54 €	RS Components	Non-specific

**Table 3:** Optional components required for operation with an external flow controller and adjustable excitation frequency.

Flow controller	Flow controller	1	1,727.00 €	1,727.00 €	Bronkhorst	Non-specific
BRIGHT unit	BRIGHT unit	1	499.00 €	499.00 €	Bronkhorst	Non-specific
PiPS		1	90.00 €	90.00 €		Non-specific
Flow_controller_mount	Flow controller mount	1	n.a.	n.a.	Your own 3D print	Polymer
BRIGHT_unit_mount	BRIGHT unit mount	1	n.a.	n.a.	Your own 3D print	Polymer
Compression nut	Compression nut	4	3.80 €	15.20 €	HPS Solutions	Metal
Stainless Nuts 1/16″	Stainless Nuts 1/16″	4	2.23 € (22.27 €)	8.91 €	BGB Analytik	Metal
Compression ring set 1/16″	Compression ring set 1/16″	4	5.10 € (50.97 €)	20.39 €	HPS Solutions	Metal
Compression ring set 3 mm	Compression ring set 3 mm	2	3.20 € (32.00 €)	6.40 €	HPS Solutions	Metal
Compression ring set 6 mm	Compression ring set 6 mm	4	2.98 € (29.76 €)	5.95 €	HPS Solutions	Metal
SS-U-ML6-NFL1-ID1-LV − FITOK Gerade Conversions-Verschraubung	SS-U-ML6-NFL1-ID1-LV − FITOK Gerade Conversions-Verschraubung	4	144.16 €	576.64 €	MF Handels GmbH	Metal
Port connector	Port connector	2	17.22 €	34.44 €	HPS Solutions	Metal
Stainless Steel Tubing	Stainless Steel Tubing	2	7.22 € (72.22 €)	14.44 €	BGB Analytik	Metal
FITOK Valve	FITOK Valve	1	113.88 €	113.88 €	HPS Solutions	Metal
C406, C407, C408, C409	CAP ALUM 1000UF 20% 50 V SMD	4	4.05 €	16.20 €	Digikey	Semiconductor
DCDC200, DCDC201	DC/DC Converter	1	13.38 €	13.38 €	Farnell	Non-specific
D200	TVS diode	1	0.19 €	0.19 €	Digikey	Non-specific
R201	2012 SMD-resistor 15 K	1	0.13 €	0.13 €	Mouser	Non-specific
R202	2012 SMD-resistor 560R	1	0.21 €	0.21 €	Mouser	Non-specific
R203	2012 SMD-resistor 1M	1	0.31 €	0.31 €	Mouser	Non-specific
R204	2012 SMD-resistor 120 K	1	0.33 €	0.33 €	Mouser	Non-specific
S201, S500	Toggle switch	1	2.69 €	2.69 €	Conrad	Non-specific
Precision Potentiometer	Precision Potentiometer	1	27.50 €	27.50 €	Mouser	Non-specific

## 6 Build instructions

### 6.1 Safety measures

This hardware is to be installed and operated only by trained personnel. During assembly, the use of a soldering iron is required, which presents a risk of burns. Exercise caution and utilize appropriate tools to prevent injury. Additionally, solder and the vapors produced during the soldering process contain substances harmful to health; therefore, it is essential to use suitable personal protective equipment (PPE). Ensure that an effective fume extraction system is in place to manage soldering fumes; these fumes should not be inhaled.

Furthermore, the pyrolysis unit is capable of operating at voltages up to 50 V. It is imperative to comply with all relevant legal regulations, as well as health and safety guidelines, to prevent electrical hazards. Ensure compliance with applicable laws to maintain safety and regulatory standards.

When handling the glass liner, always wear safety goggles and gloves to protect yourself. Handle the liner gently, as it can break easily if subjected to excessive force. Inspect it for any cracks or chips before use. Ensure that the liner is installed correctly, with proper alignment, to prevent leaks during operation. If a liner is broken, dispose of it carefully in a designated sharps container.

### 6.2 Assembly of the pyrolysis unit

**Fig. 1:**
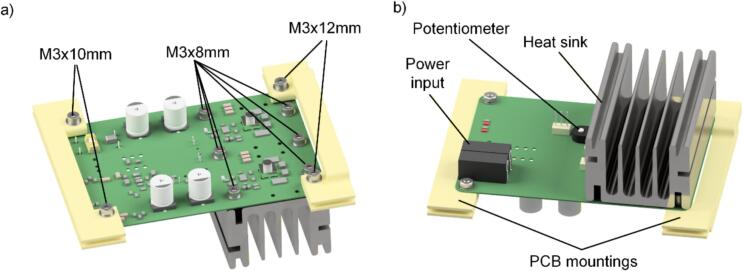
a) Top view and b) bottom view of the CAD model of the PCB with installed brackets and heat sink ready for installation in the housing.

For assembly of the pyrolysis unit, it is advisable to use the CAD model of the complete assembly. The assembly of the pyrolysis unit begins with the assembly of the electronics. The printed circuit board (PCB) should be ordered from a qualified manufacturer that meets the specifications indicated in the Gerber files. Critical parameters include the board dimensions (104 mm × 75 mm), the thickness of the PCB (1.69 mm), as well as the copper thickness (35 µm). It is advisable to utilize a stencil and solder paste for the accurate placement of surface-mounted devices (SMD). In the absence of a stencil, it is essential to first solder the smaller SMD components before progressing to larger and through-hole components. Following the assembly of the PCB, the two brackets (PCB_mount_1 and PCB_mount_2) must be 3D-printed to facilitate the installation of the PCB within the housing. A fine layer thickness, such as 0.1 mm, combined with three perimeters for wall thickness, is recommended for optimal results. For users without experience in SMD soldering, we note that several commercial prototyping services are available which offer affordable SMD assembly, even for small quantities. According to the configuration depicted in Fig. 1, the flat bracket is mounted to the side of the supply voltage and input signal connections, while the bracket with the extended section is installed on the heat sink side. Prior to installing the heat sink, it is essential to apply heat-conducting paste to the cooling surface of the GaN FETs. To prevent any potential damage to the PCB, it is recommended to just lightly tighten the heat sink screws. The use of consistent washers is recommended to ensure optimal pressure distribution. The PCB is designed to generate a square wave voltage, which is applied directly to the heating coil. The output frequency can be adjusted through a potentiometer. Through experimental testing, an optimal frequency of 500 kHz was determined, which can alternatively be set by selecting a fixed SMD resistor. For instance, setting a frequency of 500 kHz necessitates the use of a 100 kΩ resistor.

**Fig. 2:**
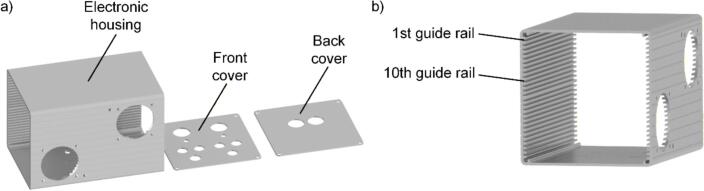
a) CAD models of the main body as well as the front and back cover of the housing by Hammond and b) a view of the main body indicating the rail to insert the PCB.

In the subsequent phase of assembly, a housing as illustrated in Fig. 2 a) is required. Therefore, several holes must be machined into the aluminum housing, as specified in the technical drawings (Enclosure.pdf, Enclosure_plate1.pdf, Enclosure_plate2.pdf). For machining the larger openings intended for fan installation, a screw hole punch is recommended. For smaller openings, a cone drill is best suited. Additionally, the two apertures designated for coil connections should be equipped with cable bushings to ensure proper insulation and protection. The connection sockets, LEDs, and additional components must be installed directly on the front panel and connected to their corresponding pads and connection points on the assembled PCB. Before inserting the PCB into the enclosure, it is essential to mount both fans onto the side of the housing and ensure their connections to the designated power supply on the PCB. Make sure that the fans are oriented correctly, with one drawing air into the housing and the other expelling it, to facilitate effective thermal management. Subsequently, the PCB should be carefully inserted into the aluminum housing along the 10th rail, as depicted in Fig. 2 b).

**Fig. 3:**
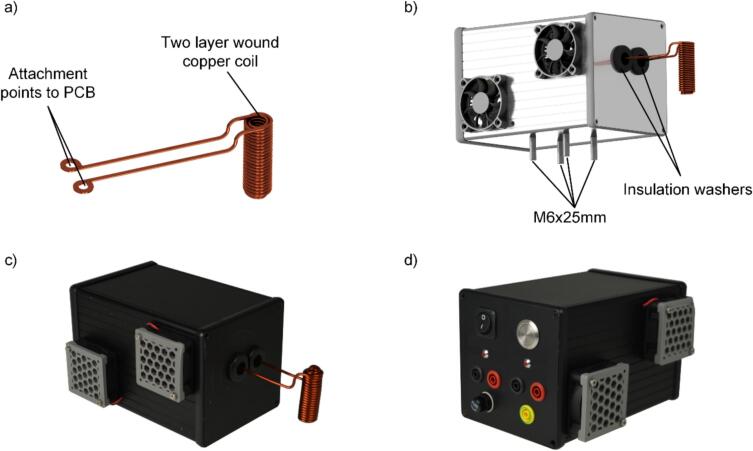
a) CAD model of the coil consisting of two layers of wound copper wire, b) CAD model of the coil mounted to the housing, c) and d) photos of the complete assembled housing, including the PCB and the coil.

The construction and installation of the heating coil is the final step in completing the electronics. Tests conducted with variously dimensioned heating coils have proven that winding the coil as tightly as feasible around the liner is beneficial. This configuration maximizes the field strength at the ferromagnetic wire by minimizing the distance between the coil and the ferromagnetic wire. Concurrently, adequate inductance is crucial for limiting inrush currents. To obtain a coil as illustrated in Fig. 3 a), a 2 mm thick copper wire has to be tightly wound around a metal rod with an approximate diameter of 7.5 mm. The coil should feature two layers, with an overall wire length of approximately 50 mm. The target inductance is approximately 2.6 µH, as indicated in the CAD model in the repository. After winding, the metal rod must be removed, and the coil ends require machining to facilitate secure attachment to the designated mounting points on the PCB. A practical method to achieve this is to bend the coil ends around an M4 screw using pliers and gently flatten them with a hammer. This enables a secure attachment of the coil to the PCB. Before finalizing the housing assembly, insert four M6x25 screws with washers through the pre-drilled holes in the base of the housing as shown in Fig. 3 b). These screws will be used to mount the enclosure to the base plate of the pyrolysis unit. Moreover, one of the four screws should be equipped with a cable lug to establish an earth connection. Fig. 3 c) and d) show the complete assembled housing.

**Fig. 4:**
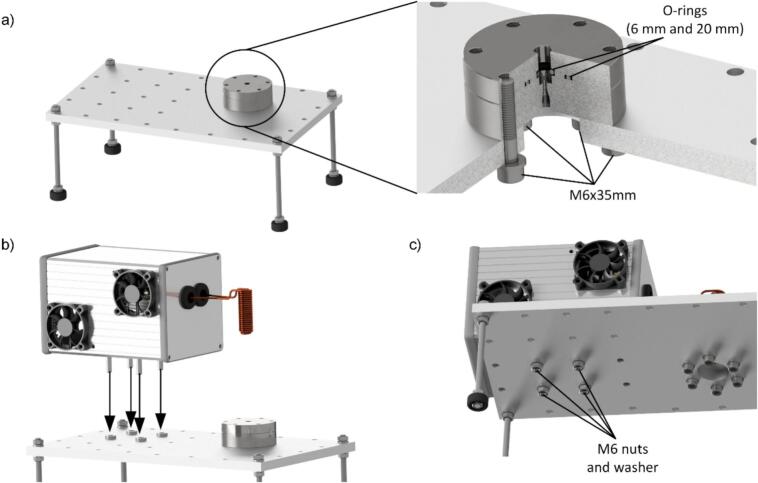
a) CAD model of the bottom plate with installed feet and detailed view of the bottom flange of the pyrolysis chamber mounted using six M6x35 mm screws, b) and c) installation of the electronics on the bottom plate fixed by four M6x25 mm and corresponding nuts.

Following the assembly of the electronics, the mechanical components of the pyrolysis unit must be assembled. This requires attaching the rubber bumpers to the threaded rods, as these will serve as feet when they are screwed into the base plate, as shown in Fig. 4 a). Afterwards, mount the bottom adapter to the bottom plate using the bottom flange as well as six M6x35 mm screws and the appropriate washers, as shown in detail in Fig. 4 a). It is crucial to ensure that the two O-rings (with diameters of 6 mm and 20 mm, respectively) are installed into their designated grooves to ensure a gas-tight seal. Subsequently, the electronics box can be secured to the bottom plate using four M6x25 mm screws and corresponding nuts as shown in Fig. 4 b) and c). To maintain proper spacing, insert two washers between each screw, acting as spacers, to separate the housing from the bottom plate.

**Fig. 5:**
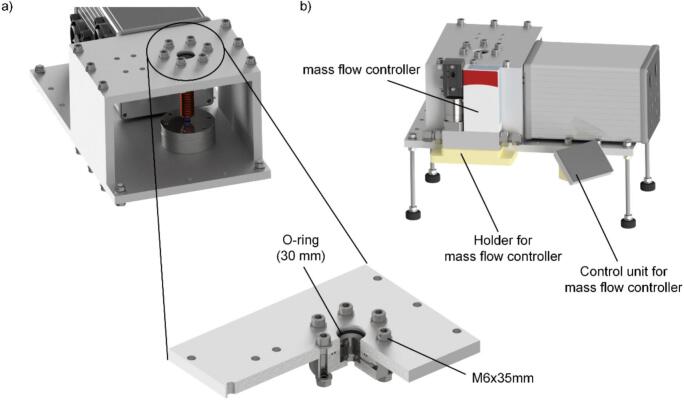
a) Detailed view of the top plate of the pyrolysis unit and b) side view of the pyrolysis unit with mass flow controller and BRIGHT unit mounted. © Bronkhorst High-Tech B.V. [2025]. Used with permission.

Following the previous steps, secure the top adapter to the top plate with six M6x35 screws, ensuring the use of washers and nuts for firm attachment, as shown in Fig. 5 a). An O-ring with a 30 mm internal diameter must be precisely positioned within the adapter's groove to ensure proper sealing. The adapter is designed with two gas line connections, though only one is utilized for the nitrogen supply. The second connection is available for potential pressure regulation. Proceed by installing the two side panels to the bottom plate using three M6x25 screws each. Optionally, at this stage, the mount for the mass flow controller (MFC) can be 3D printed and subsequently attached to one of the side panels using M6x35 screws, as shown in Fig. 5 b). The MFC should be mounted using two M4x20 screws. For the gas line connection from the MFC to the pyrolysis unit, it is recommended to employ a stainless-steel capillary with appropriate Vici connectors, as specified in the parts list. Finally, secure the ceiling panel to the side panels. If an MFC is integrated into the setup, the bracket for the BRIGHT unit can be 3D printed and installed adjacent to the electronics housing using two M6x40 screws.

**Fig. 6:**
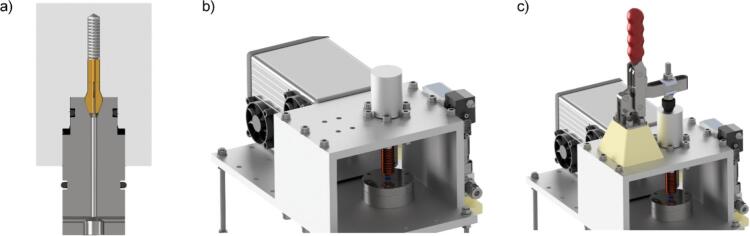
a) Detailed cut view of the CAD model of the knob, b) CAD model showing the liner and knob installed to the pyrolysis, and c) toggle clamp(©RS Components) mounted on the 3D-printed fastening block.

Proceed with assembling the knob by first placing the spring and collet into the designated hole in the outer handle knob, as shown in detail in Fig. 6 a). Following this, insert a 12 mm O-ring into the upper groove of the inner knob part, and a 17 mm O-ring into the lower groove. As indicated in Fig. 6 b), the liner must be guided through the top adapter and the coil positioned within the bottom adapter before inserting the assembled handle into the pyrolysis unit. Given the narrow 6 mm diameter of the lower O-ring, it may be advantageous to apply a small amount of isopropanol or a similar lubricant to facilitate the insertion of the liner. Ensure that the isopropanol has completely evaporated before initiating any measurements or operations to avoid interference or potential safety issues. As shown in Fig. 6 c), the 3D-printed mounting block is required to securely attach the toggle clamp. Due to the block absorbing the forces generated during clamping, it is recommended to use a high infill percentage, ideally 50% or greater, to ensure structural integrity. Once printed, the block should be mounted to the ceiling plate using four M6x70 screws, along with the appropriate washers and nuts to provide stability and strength. The M8 screw with the toggle clamp should be equipped with the corresponding rubber cap to ensure a secure and gentle contact. This assembly should exert pressure centrally on the handle to secure it within the pyrolysis unit. The pressure applied to the handle can be finely adjusted by modifying the height of the M8 screw, allowing for precise control of the clamping force.

The assembly of the pyrolysis unit requires careful attention to the outlined instructions to ensure optimal performance and safety. From the installation of electronics and power connections to the integration of mechanical components, such as the heating coil and handle, each step is designed to maintain the structural integrity and functionality of the device.

## 7 Operation instructions

**Fig. 7:**
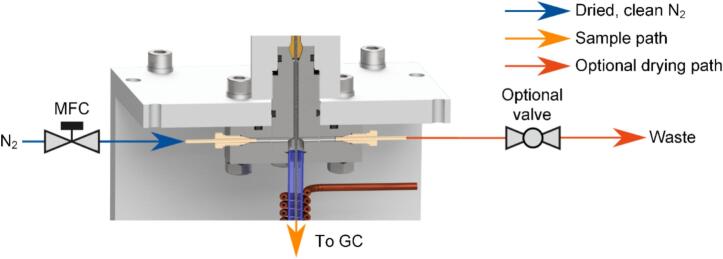
Schematic representation of the gas flow when using an optional valve at the second gas port at the upper adapter for drying moist samples before beginning the pyrolysis.

To operate the pyrolysis unit, the outlet must first be connected to the inlet of the subsequent measuring system, *e.g.*, GC. It is essential to ensure a completely gas-tight connection, as the products generated during pyrolysis can be harmful to health. Next, a nitrogen flow of 50 ml/min should be provided. An MFC (e.g., Bronkhorst FG-201CV) is well-suited for this purpose and can be connected to one of the two gas ports of the upper adapter, while the remaining gas port has to be sealed gas-tight for basic operation. However, the MFC is not part of the pyrolysis unit and can be substituted with a simple needle valve or a flow restriction to reduce costs. When measuring biological samples with the pyrolysis unit, it is most likely that the sample contains considerable amounts of water left over from the dilution step or the sampling process, which ideally must be removed before the actual measurement. As shown in Fig. 7, the remaining gas port can therefore be equipped with an optional valve to direct the gas flow directly to a fume hood during the drying phase, before switching the gas flow to the GC column for analysis.

**Fig. 8:**
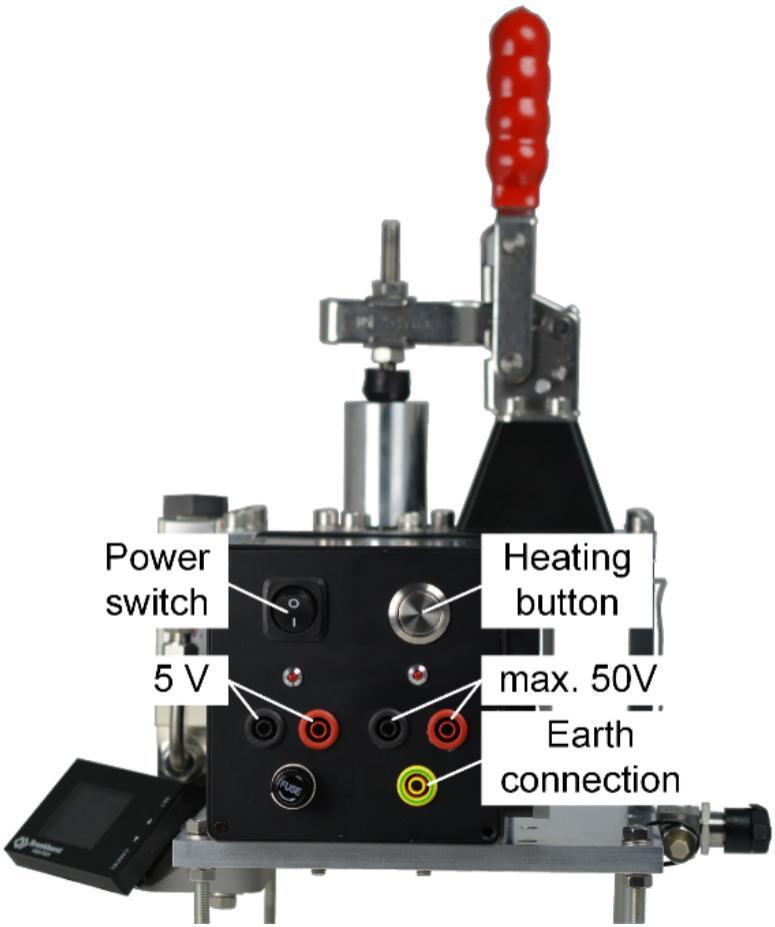
Photo of the overall assembly with a view of the electronics housing, showing the labeled power supplies for the electronics (5 V) and for the heating process (max. 50 V).

Before operating, it is crucial to ground the entire pyrolysis unit by connecting an appropriate cable to the Earth connection terminal before applying power. As shown in Fig. 8, two power supplies are required to run the pyrolysis unit. Firstly, a 5 V voltage supply is necessary for the electronics and must be present throughout the entire operation cycle. Additionally, to supply the heating of the ferromagnetic wire, at least 20 V is required. Otherwise, the ferromagnetic wire will not reach its Curie temperature. The maximum voltage is 50 V and must not be exceeded, as it may destroy the electronics.

Subsequently, the ferromagnetic wire, which serves as the sample holder, is prepared. The Curie temperature of the ferromagnetic wire depends on its material composition. For the measurements conducted later, a ferromagnetic wire made of an iron-nickel alloy (Fe50Ni50) was used, which has a Curie temperature of 520 °C. However, other alloys with different Curie temperatures can also be used, depending on the specific application. Safety and hazard warnings provided by manufacturers must always be observed, as certain components, such as nickel, can be potentially hazardous to health. The wire should have a diameter of 1 mm and be cut to a length of 13.8 mm to be inserted into the handle. It is mandatory to ensure it is mechanically fixed and cannot slip out. The ferromagnetic wire must be free from impurities before applying the sample, as any residues can distort the measurement results. An effective cleaning method involves heating the ferromagnetic wire in a vacuum furnace or baking it inside the pyrolysis unit using a 20 V supply voltage for several minutes. Turn on the pyrolysis unit by switching the power switch to the “1″ position. In this state, the pyrolysis unit is ready to perform measurements, but the ferromagnetic wire is not yet heated. A heating cycle of the pyrolysis unit is run by pressing the heating button. The heating voltage is applied as long as the heating button is pressed.

To ensure optimal reproducibility, the sample should be available in liquid form. For solid samples, it is advisable to dissolve them in a neutral solvent (e.g., ultrapure water). To apply the sample, the ferromagnetic wire is dipped into the liquid, and due to surface tension, a small amount of the sample adheres to the surface of the ferromagnetic wire. It should be noted that, depending on the subsequent measuring system, even the smallest amounts of sample can be sufficient. In our case, an immersion depth of approximately 5 mm was found to be ideal. However, this strongly depends on the sample and solvent and needs to be adapted to the application and the subsequent analytical method. Subsequently, the handle is inserted into the pyrolysis unit and secured with the toggle clamp before the pyrolysis process can begin. The duration to reach the Curie temperature of the ferromagnetic wire depends on the applied supply voltage. The power consumption peaks at the start of the heating process and quickly falls to a steady state. For example, the power consumption at a supply voltage of 20 V is initially approximately 10 W and remains at about 7 W in the steady state. At 40 V, the corresponding values are 35 W and 16 W, respectively. In the following exemplary measurements, a pyrolysis duration, including the heating process to the Curie temperature, of 6 s proved to be a good value.

Despite its low thermal mass, the ferromagnetic wire remains hot even after the heating process is complete and therefore requires extreme caution when handling. It is strongly advised to allow the ferromagnetic wire to cool before removing the handle from the pyrolysis unit. After the measurement, the ferromagnetic wire is contaminated with pyrolysis product residues. Generally, it is best to dispose the ferromagnetic wire after each measurement and replace it with a new one. However, if the application of the measurement does not require extreme sensitivity, a cleaning in an ultrasonic bath seems also reasonable.

## 8 Validation and characterization

Testing of the pyrolysis unit started with the investigation of the temperature rise of the ferromagnetic wire using an optical temperature sensor Optris (CTLaser 3MH1 with CF2 lens). This optical temperature sensor provides a measurement spot of less than 0.5 mm, fast sampling rate of 1 kHz, and is capable of measuring temperatures from 150 °C upwards. Thus, to determine the rise time of the temperature of the ferromagnetic wire, extrapolation of the temperature course for temperatures below 150 °C is required. The rise time of the temperature of the ferromagnetic wire was determined using the so-called 10/90 rise time method. During the heating process, the temperature was measured in real-time using the optical temperature sensor. The stationary end temperature was determined by averaging over 1 s (1000 data points), when the slope of two consecutive moving averages of 1 s falls below 10^-3^ K/s for the first time. However, as the optical temperature sensor can only measure temperatures above 150 °C, the exact temperature profile of the ferromagnetic wire between ambient temperature and 150 °C remains unknown. To address this issue, a second linear fit is calculated using the first 300 data points after the 150 °C mark is exceeded. This second linear fit allows for an approximation of the time at which the ferromagnetic wire reaches 10% of the temperature difference. Since the temperature curve exhibits the steepest slope at the beginning of the heating process and only slightly levels off as temperature increases, this approach is likely to overestimate rather than underestimate the rise time. To ensure accurate results regarding the rise time of temperature, reproducibility, and stability of the final temperature, the optical temperature sensor is precisely aligned before each measurement series. Therefore, initially, a coarse alignment is performed. Afterwards, the heating process is activated, and the ferromagnetic wire is heated to a stationary end temperature. Fine adjustments to the sensor mount are performed until the measured temperature reaches a maximum, ensuring the lens's focal point is precisely centered on the ferromagnetic wire. To ensure that the ferromagnetic wire is at the same temperature before each measurement run, it was immersed in water to cool it back to room temperature.

**Fig. 9:**
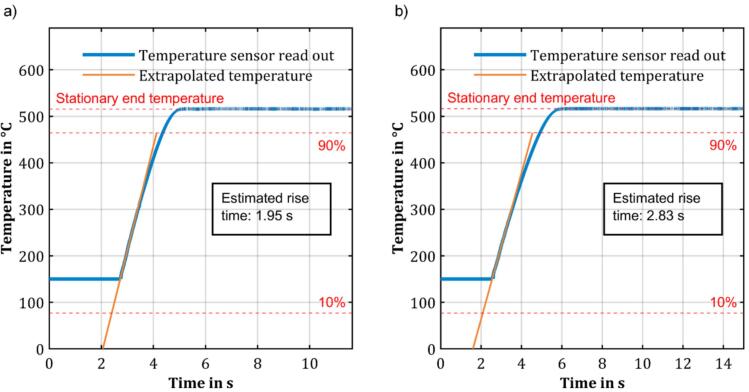
Rise time of the temperature of the ferromagnetic wire inside the pyrolysis chamber with the coil excited with 24 V and a frequency of a) 340 kHz and b) 500 kHz, measured with the optical temperature sensor.

Fig. 9 shows the measured temperature profiles at excitation frequencies of 340 kHz and 500 kHz over a period of 10 s with the emissivity coefficient of the optical temperature sensor set to 0.564. The heating voltage of 24 V resulted in an RMS value of the coil current of approximately 14.8 A and 10.2 A during the heating process, respectively.

**Fig. 10:**
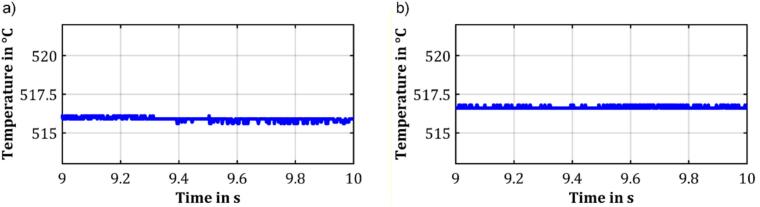
Stability of the stationary end temperature for an excitation of the coil using a) 340 kHz and b) 500 kHz and a voltage of 24 V.

In both measurements, the temperature curve initially rises steeply, then levels off, and remains at a constant level. The measured stationary end temperatures are 516.6 °C and 515.9 °C, respectively. The small deviation of the stationary end temperature from the Curie temperature of the used ferromagnetic wire is most likely attributed to slight differences between the emissivity coefficient used and the actual emissivity coefficient of the ferromagnetic material. The close proximity of the stationary end temperatures of the two measurements appears plausible, as the underlying physical process limits the final temperature and is independent of frequency within the specified frequency range. Both in the first and second measurement, the stationary end temperature proves to be very stable with a standard deviation of the last 1000 recorded data points of 0.11 K and 0.08 K, respectively. It should be noted that the sensor noise may negatively affect these values, especially since they fall within the optical temperature sensor's resolution of 0.1 K. Therefore, it can be assumed that the actual stability of the temperature of the ferromagnetic wire is even better. A detailed excerpt of these last 1000 data points is shown in Fig. 10. Furthermore, it is noticeable that the stationary end temperatures are approximately in the vicinity of 520 °C, indicating that the emissivity coefficient has been chosen adequately.

**Fig. 11:**
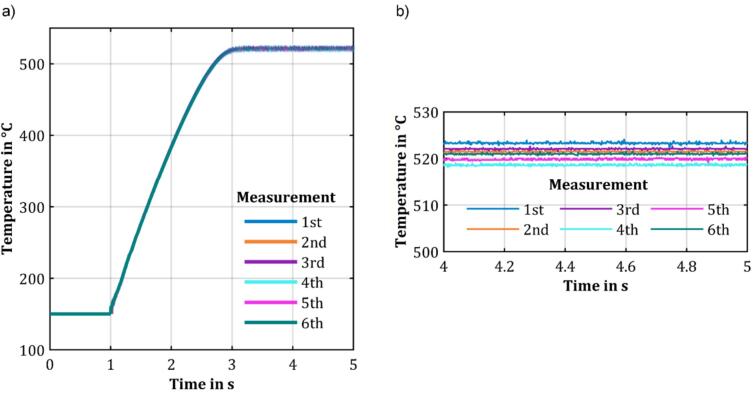
a) Repeatability of the heating process of the ferromagnetic wire and b) reproducibility of the stationary end temperature.

Fig. 11 illustrates a series of six measurements to evaluate reproducibility of the heating process of the pyrolysis unit. For this purpose, temperature profiles were recorded at an excitation frequency of 340 kHz with a heating voltage of 24 V. The individual temperature profiles are overlaid at the point where the 150 °C was first exceeded. The slopes of all six temperature profiles are nearly identical, with only slight deviations in the stationary end temperatures. The biggest difference in stationary end temperature is between the first and fourth measurements, accounting for 4.7 K, while the standard deviation of all six stationary end temperatures is just 1.7 K, corresponding to approximately 0.2% maximum deviation with respect to the stationary end temperature. This is also within the range of the optical temperature sensor's repeatability accuracy, which is approximately 1.5 K. The slightly higher deviation might be attributed to a slight shift in the position of the ferromagnetic wire between measurements, as the handle, along with the ferromagnetic wire, was removed from the housing after each measurement for cooling. This is supported by the fact that the stationary end temperature of the first measurement is the highest, as the sensor was precisely aligned to the ferromagnetic wire before the first measurement and not realigned in the following measurements.

**Fig. 12:**
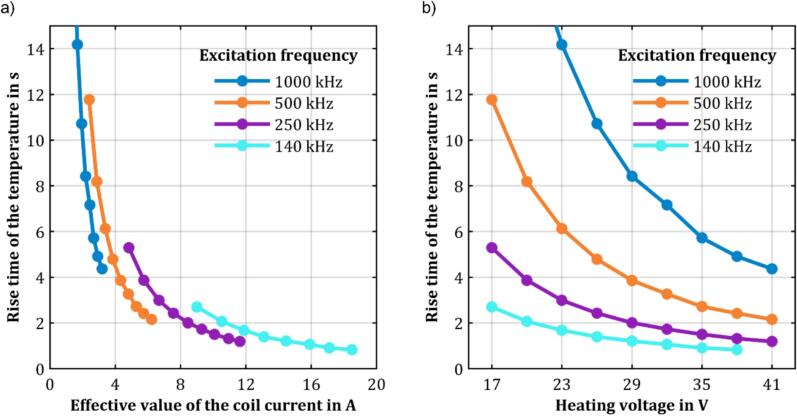
Dependence of the rise time of the temperature on a) the root mean square value of the coil current and b) the heating voltage at different excitation frequencies.

To understand the relationship between the rise time of the ferromagnetic wire temperature and the coil current, as well as the heating voltage, variations of these two parameters were investigated. Initially, the frequency range was extended to 140 kHz to 1 MHz using a potentiometer with greater resistance, allowing measurements at lower frequencies. For these measurements, the heating voltage was increased in steps of 3 V, from 17 V to 41 V, at various frequencies, while simultaneously measuring the coil current and the rise time of the ferromagnetic wire temperature. The results of these measurement series are presented in Fig. 12. As can be seen, the rise time of the ferromagnetic wire temperature decreases continuously as the coil current and heating voltage increase. However, the rate of decrease in the rise time of the ferromagnetic wire temperature diminishes at higher current or voltage values, causing the curves to asymptotically approach the x-axis. Additionally, it is apparent that the rise time of the ferromagnetic wire temperature decreases with increasing frequency at a constant coil current. This observation is consistent with the theory that hysteresis losses increase with frequency at a constant current. For constant heating voltage, it can be concluded that the minimum rise time of the temperature of the ferromagnetic wire occurs at a frequency of 140 kHz. However, as lower frequencies generally lead to higher coil currents, which in turn result in higher losses and, thus, higher temperatures in the MOSFETs, reducing their lifetime, a frequency of 500 kHz was used for the following measurements.

To showcase one exemplary application, the pyrolysis unit was coupled to a GC-IMS to test different biological samples. The GC-IMS consists of an Agilent 7890A (Agilent, USA) GC and an ultra-fast polarity switching IMS, as shown in [26]. A Restek Rxi-5Sil MS 30 m GC column (with an inner diameter of 560 µm and a film thickness of 1.5 µm) with a nitrogen carrier gas flow of 5 ml/min was used. For the GC measurements, a GC temperature ramp was applied from 40 °C to 240 °C over 12 min, with additional periods of constant temperature of 2 min at both the start and end of the GC run. Measurements with biological samples were carried out using probiotics OMNi-BiOTiC Panda containing Lactococcus lactis W58, Bifidobacterium lactis W51, Bifidobacterium lactis W52 and *Bifidobacterium bifidum* W23. The probiotics were dissolved in 120 ml of lipid-free, ultra-pure water (TMS-011, Sigma-Aldrich). From this solution, 500 µl was transferred into a vial to maintain a consistent sample volume and ensure the same small amount was applied to the ferromagnetic wire. After homogenizing the solution by gentle swirling, the ferromagnetic wire from the pyrolysis unit was immersed in the probiotic solution and stirred gently for about 3 s. The ferromagnetic wire was then transferred to the pyrolysis unit for a 10-minute pre-heating phase for drying the sample. Subsequently, the GC temperature ramp and the pyrolysis sequence were started simultaneously.

**Fig. 13:**
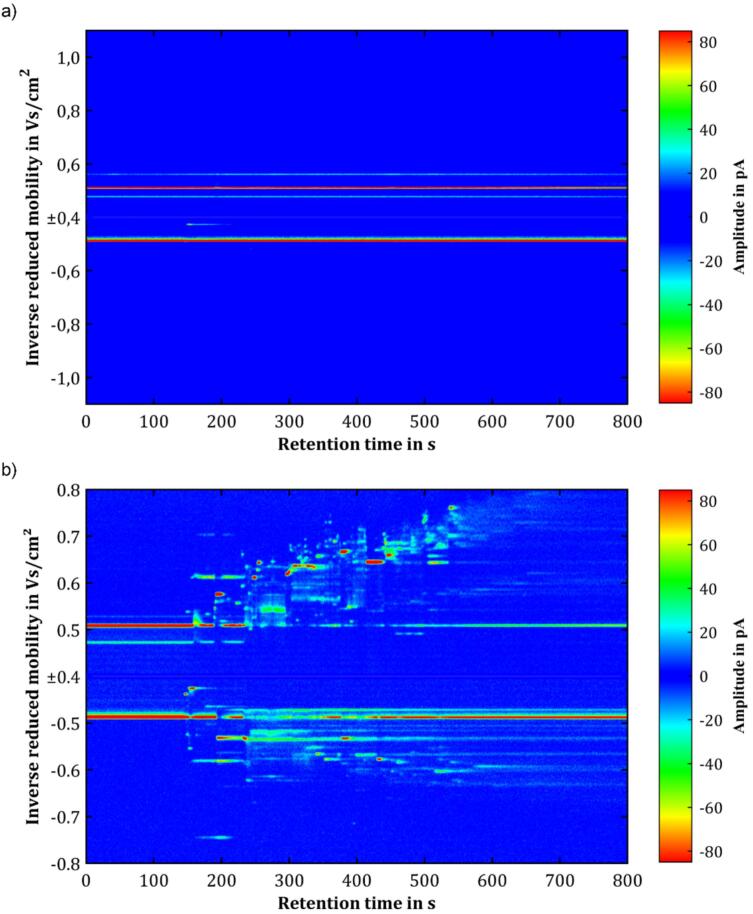
Topographic plots of the Pyrolysis-GC-IMS data of a) a blank measurement and b) a measurement with a sample containing probiotic OMNi-BiOTiC Panda.

Fig. 13 shows the topographic plots of a blank measurement and a measurement with a sample containing the probiotic OMNi-BiOTiC Panda. As expected, the blank measurement primarily shows the reactant ion peaks of both the dominating positive reactant ions ($1/K_{0}=0.51Vs/cm^{2}$) and the dominating negative reactant ions ($1/K_{0}=0.49Vs/cm^{2}$) of the IMS. Only a few other signals were detected, which can be attributed to minor contamination of the pyrolysis components or the ferromagnetic wire. However, as IMS is an extremely sensitive technology, those slight contaminations are most likely in a concentration range of ppb_v_ or even ppt_v_. Thus, the pyrolysis unit can be considered suitable for use in the analytical field. In contrast, when adding a sample containing probiotics OMNi-BiOTiC Panda, a large number of signals can be detected in both ion polarities. In addition, the majority of these signals exhibit significantly higher intensities compared to the blank measurement, indicating they are likely present in higher concentrations than the contaminants seen in the blank measurement. In this context, it is important to note, that these exemplary measurements are just meant to demonstrate functionality of the developed pyrolysis unit. Identification the pyrolysis products, *e.g.*, by GC–MS or GC-IMS-MS, or further interpretation of the data are not within the scope of this work.

## 9 Conclusion

To conclude, this paper presents a pyrolysis unit based on the principle of the Curie temperature. It is capable of pyrolyzing samples with variable heating rates of up to 1.9 s, depending on the excitation frequency of the coil and the voltage of the heating supply. The stationary end temperature depends on the material properties of the ferromagnetic wire and can typically be selected between 350 °C and 1100 °C. The pyrolysis unit can be easily coupled to commercially available GCs, which makes it suitable for many applications and detection techniques. With its modular, low-cost and compact design, the device is not only suitable for laboratory research but also shows strong potential in other applications, including on-site quality control, rapid screening, and educational purposes. Its compatibility with different analytical platforms further broadens the scope for integration into existing workflows. In this work, coupling to GC-IMS was demonstrated using probiotic samples. While detailed analysis of pyrolysis products (e.g., using GC–MS or GC-IMS-MS) was beyond the scope of this study, future work will extend the application of this unit to the determination of a wide range of target compounds across various sample matrices. Specifically, in our next study, we aim to investigate and differentiate various bacterial species based on their distinct pyrolysis fragment patterns using Py-GC-IMS. Moreover, ongoing developments may further improve the device’s automation and throughput, facilitating its use in routine and industrial settings. For commercial use, extensive testing, proper housing and hard- and software interfaces to existing instruments would be required, which, however, goes beyond the scope of this work.

## Ethics statements

Nothing to declare.

## CRediT authorship contribution statement

**Martin Lippmann:** Writing – original draft, Validation, Resources, Methodology, Formal analysis, Conceptualization. **Moritz Hitzemann:** Writing – review & editing, Validation, Resources, Methodology, Investigation, Formal analysis, Conceptualization. **Ayke Eckhardt:** Writing – review & editing, Visualization, Validation, Software, Resources, Investigation, Formal analysis, Data curation. **Daniel Röckrath:** Writing – review & editing, Validation, Investigation. **Stefan Zimmermann:** Writing – review & editing, Supervision, Resources, Project administration, Funding acquisition, Conceptualization.

## Declaration of competing interest

The authors declare that they have no known competing financial interests or personal relationships that could have appeared to influence the work reported in this paper.
